# 

**Published:** 1886-07

**Authors:** 


### A Monthly Journal

DEVOTED TO

### DENTAL AND ORAL SCIENCE.

W. C. BARRETT, M. D., D. D. S.,

EDITOR.

The New York Dental Journal Association,

PUBLISHERS,

No. 35 West 46th Street, New York.

AND

No. 208 Franklin St., Buffalo, N. Y.

$2.50 Per Annum in Advance, . . . . Single Copies, 25 Cents.

Entered at the Post-Office, Buffalo, N. Y., as Second-Class matter.

